# High Frequency Ultrasound for In Vivo Pregnancy Diagnosis and Staging of Placental and Fetal Development in Mice

**DOI:** 10.1371/journal.pone.0077205

**Published:** 2013-10-14

**Authors:** Adelaide Greco, Monica Ragucci, Anna Rita Daniela Coda, Alessandro Rosa, Sara Gargiulo, Raffaele Liuzzi, Matteo Gramanzini, Sandra Albanese, Sabina Pappatà, Marcello Mancini, Arturo Brunetti, Marco Salvatore

**Affiliations:** 1 Dipartimento di Scienze Biomediche Avanzate, Università degli studi di Napoli Federico II, Napoli, Italy; 2 IRCCS Fondazione SDN, Napoli, Italy; 3 Istituto di Biostrutture e Bioimmagini, CNR, Napoli, Italy; 4 Ceinge, Biotecnologie Avanzate, scarl, Napoli, Italy; Medical Faculty, Otto-von-Guericke University Magdeburg, Medical Faculty, Germany

## Abstract

**Background:**

Ultrasound is a valuable non-invasive tool used in obstetrics and gynecology to monitor the growth and well being of the human fetus. The laboratory mouse has recently emerged as an appropriate model for fetal and perinatal studies because morphogenetic processes in mice exhibit adequate homology to those in humans, and genetic manipulations are relatively simple to perform in mice. High-frequency ultrasound (HFUS) has recently become available for small animal preclinical imaging and can be used to study pregnancy and development in the mouse. The objective of the current study was to assess the main applications of HFUS in the evaluation of fetal growth and placental function and to better understand human congenital diseases.

**Methodology/Principal Findings:**

On each gestational day, at least 5 dams were monitored with HFUS; a total of ∼200 embryos were examined. Because it is not possible to measure each variable for the entire duration of the pregnancy, the parameters were divided into three groups as a function of the time at which they were measured. Univariate analysis of the relationship between each measurement and the embryonic day was performed using Spearman’s rank correlation (Rs). Continuous linear regression was adopted for multivariate analysis of significant parameters. All statistical tests were two-sided, and a p value of 0.05 was considered statistically significant.

**Conclusions/Significance:**

The study describes the main applications of HFUS to assess changes in phenotypic parameters in the developing CD1 mouse embryo and fetus during pregnancy and to evaluating physiological fetal and placental growth and the development of principal organs such as the heart, kidney, liver, brain and eyes in the embryonic mouse. A database of normal structural and functional parameters of mouse development will provide a useful tool for the better understanding of morphogenetic and cardiovascular anomalies in transgenic and mutant mouse models.

## Introduction

Real-time ultrasound imaging is routinely used for assessing pregnancy, fetal development and well being, as well as the effects of treatments that could alter these parameters in humans [1], [2]. Common measurements performed by ultrasound to evaluate human fetal growth are outer–outer biparietal diameter (BPD), head circumference (HC), mean abdominal diameter (AD), abdominal circumference (AC) and femur length (FL) [1]. The laboratory mouse has emerged as an appropriate model for fetal and perinatal studies because morphogenetic processes in mice show adequate homology to those in humans. Moreover, mice are inexpensive, are easy to handle and house, and are often used for genetic manipulations. CD1 is the outbred laboratory mouse strain that is most widely used in basic reproductive biology studies in which genetic variability in the experimental population is desirable. In developmental studies, it is highly desirable to acquire longitudinal data in a non-invasive manner and with high spatial and temporal resolution and to define structural parameters [1]–[2]. Several imaging approaches have become available for the study of mouse development, including ultrasound, magnetic resonance, optical imaging and confocal biomicroscopy [3]. Advances in ultrasound technology have recently led to the development of high-frequency ultrasound (HFUS), which can be used for the high-resolution imaging of mouse embryonic development [4]–[10]. HFUS is a non-invasive, relatively safe and inexpensive molecular imaging technology.

Since the first study of mouse embryos in utero was performed by Turnbull et al. in 1995 [11], ultrasound has been used for both transcutaneous and transuterine imaging. Some studies employed conventional 13–15 MHz ultrasound systems [12], with spatial resolutions ranging from 200 to 500 µm. High-frequency systems (40–100 MHz), which offer a spatial resolution of approximately 30 µm and penetration depths of several millimeters, are more often used for mouse embryo imaging. These sound waves are generally harmless at reasonable doses, particularly in the soft tissue environment of the embryo. Many studies have also reported the use of Doppler ultrasound for measuring the direction and velocity of blood flow. The change in the frequency of sound waves as they reflect off moving blood cells produces a Doppler effect, and the shift in frequency between the transmitted and received waves can be used to calculate the velocity of the moving blood [3], [13]. HFUS appears to be a practical tool that can be used to determine gestational age, to monitor placental and fetal growth and embryonic or fetal viability, to evaluate morphological development, and to assess physiological cardiovascular structure in the mouse embryo and fetus throughout pregnancy. Its use would also contribute to improved animal welfare, in accordance with the philosophy of the 3 Rs model of Russell and Burch (Refinement, Reduction and Replacement). Early confirmation of pregnancy or fetal wastage in valuable animals can be used as evidence for delayed development or growth retardation in mutant or manipulated animals compared with wild-type animals [5]. Transcutaneous images of implantation sites can be obtained in pregnant mice at 5 days of gestation. At embryonic day (E) 6.5, implantation sites are clearly visible as regions in which the cross-section of the uterus is enlarged, and a small, echo-free region, which is likely the proamniotic cavity of the developing embryo, is detectable within the center of each enlargement. By E7.5, three dark regions are visible within the conceptus, corresponding to the ectoplacental, amniotic and exocoelomic cavities of the developing embryo. At E 8.5, the embryo and amniotic membrane are visible, and the allantois can be observed emerging from the embryo and approaching the ectoplacental cone, where the chorioallantoic placenta later develops. On day 9.5, the amniotic membrane, amniotic and yolk sac cavities, brain, cerebral ventricles and heart are visible. HFUS allows the accurate characterization of morphological details specific to each stage of development. Over the past several years, a number of authors have demonstrated the effective use of ultrasound backscatter microscopy to evaluate the development of the central nervous system, eye, and heart in fetal mice [14]–[18]. Later during embryonic development, many other structures can also be visualized using transcutaneous imaging, including the developing paw and forelimb, eyes, lung, liver, kidney, vertebrae, and veins. Limb buds are first apparent at E10.5, and individual hind- and forelimb digits become discernible at E15.5. The spine first appears as a faint echogenic line at E12.5. Ossification of the vertebral elements, the face and skull bones, and ribs can be identified at E13.5 and becomes progressively more evident, more echogenic, and more defined. The echogenic lungs grow increasingly distinct from the more hypoechoic liver beginning at approximately E12.5. The fetal stomach and urinary bladder are somewhat identifiable beginning at E13.5, when they appear as hypoechoic structures [6].

The survival and growth of the fetus are critically dependent on the placenta. Placental growth is often neglected despite this critical role, and abnormal placental size is now recognized as an early predictor of poor fetal growth and poor outcome in human pregnancies. The mouse has been widely used as an animal model to better understand physiological human implantation and placentation and anomalies in these processes, owing to its significant morphostructural and histological homologies to the same features in humans [19], [20]. Anomalous intrauterine growth has been defined as a critical risk factor for perinatal and childhood morbidity and mortality and for diverse adult-onset diseases including diabetes, cancer, and hypertension. We have used HFUS for the diagnosis of pregnancy and the staging of placental and fetal development in CD1 mice. Statistical analysis was performed to assess changes in embryonic and placental morphology in relation to the embryonic day. These data will permit the accurate staging of pregnancy, which is necessary if therapy is to be performed at a precise time and if morphological abnormalities are to be identified at specific embryonic stages in the evaluation of animal models of congenital diseases.

## Materials and Methods

Animal studies were performed according to a protocol approved by the Italian National Institutes of Health and the Animal Welfare Regulation Committee of the University Federico II of Naples, in adherence to the Guide for the Care and Use of Laboratory Animals published by the US National Institutes of Health (NIH Publication No. 85-23, revised 1996). Thirty CD1 adult female mice were analyzed by HFUS during pregnancy from E6.5 to E16.5 to evaluate different morphometric parameters. At each gestational day, at least 5 dams were monitored, for a total of ∼200 embryos examined. To reduce the number of animals needed for experimental purposes, more than one measurement was made in each embryo, with caution taken to limit the duration of anesthesia (Table 1). Embryonic day E0.5 of pregnancy was estimated by the presence of a vaginal plug after overnight mating.

**Table 1:** Number of embryos for each measurements.

Type of measurements	Gestational Age (Day)	Number of measured embryos
Decidual thickness	E6.5–9.5	95
Gestational sac length	E7.5–11.5	112
Gestational sac thickness	E7.5–11.5	112
Implantation site length	E6.5–E9.5	95
Implantation site thickness	E6.5–E9.5	95
Placental length	E9.5–E16.5	128
Placental thickness	E9.5–E16.5	128
Crown-rump length	E7.5–E14.5	152
Abdominal circumference	E11.5–E16.5	81
Antero-posterior abdominal diameter	E11.5–E16.5	81
Latero-lateral abdominal diameter	E11.5–E16.5	81
Occipital-snout length	E12.5–E16.5	61
Biparietal diameter	E9.5–E16.5	126
Subretinal space thickness	E12.5–E16.5	68
Antero-posterior lens diameter	E12.5–E16.5	68
Latero-lateral lens diameter	E12.5–E16.5	68
Stomach diameter	E12.5–E16.5	59

During the ultrasound examination, pregnant mice were lightly anesthetized using isoflurane (4% induction dose, 2.0-1.5% maintenance dose) plus oxygen (1 L/min). The mean anesthesia time increased with gestational age (range 20–45 min/dam) because the scanning time was increased to accommodate the greater number of measurements that could be recorded as the fetuses matured. Body temperature was monitored with a rectal probe and maintained in a physiological range using an infrared lamp. Hair was removed from the dam’s abdomen by shaving, followed by the use of a chemical hair remover; then, ultrasound gel was applied to the skin to facilitate sound transmission and to reduce contact artifacts.

Embryos were imaged through the maternal abdominal wall with a Vevo 770 instrument (VisualSonics, Canada). The 40 MHz high-resolution linear transducer was used (focal length 6 mm, depth of penetration 5–15 mm; resolution, 30–40 µm axial and 70–90 µm lateral). Two to four embryos were imaged in a single session to avoid prolonged anesthesia. Images of implantation sites, developing organs and quantitative variables for monitoring fetal growth were obtained in cross-sectional two-dimensional mode (B-mode). All measurements were made by the same operator to ensure consistency during the experiment and to avoid excessive consecutive observations, which might damage the dam or its conceptus.

## Statistical Analysis

Because it is not possible to measure each variable for the entire duration of pregnancy, the parameters were divided into three groups as a function of the time at which they were measured.

Group 1, from E7.5 to E9.5: decidual thickness, gestational sac length, gestational sac thickness, implantation site length, implantation site thickness, and crown-rump length;

Group 2, from E9.5 to E11.5: gestational sac length, gestational sac thickness, placental length, placental thickness, crown-rump diameter, and biparietal diameter; and

Group 3, from E12.5 to E16.5: placental length, placental thickness, abdominal circumference, antero- posterior abdominal diameter, latero-lateral abdominal, diameter, occipital-snout length, biparietal diameter, subretinal space thickness, antero-posterior lens diameter, latero-lateral lens diameter, and stomach diameter.

Univariate analysis between each measurement and embryonic day was performed using Spearman’s rank correlation (Rs). Continuous linear regression was adopted for multivariate analysis of significant parameters. All statistical tests were two-sided, and a p value of 0.05 was considered statistically significant. Statistical analyses were performed using MedCalc 12 (Ostend, Belgium).

## Results

High-frequency ultrasound (HFUS) is a valuable tool for monitoring placental and fetal growth. From E6.5 to E15.5, at least 5 dams were examined on each embryonic day. At E16.5, only 2 dams were examined, and few qualitative and quantitative evaluations were performed. Morphometric evaluations were conducted from E6.5 to E16.5. The values collected at several gestational ages (mean ± SD) are reported in Table 2.

**Table 2:** Morphometric evaluation.

Type of measurement	Measurement at first day (mean ± SD)	Measurement at last day (mean ± SD)
Decidual thickness	1,01±0,11 mm (E6.5)	0,94±0,22 mm (E9.5)
Gestational sac length	0,90±0,27 mm (E7.5)	5,28±1,52 mm (E11.5)
Gestational sac thickness	0,62±0,36 mm (E7.5)	3,22±0,70 mm (E11.5)
Implantation site length	3,66±0,24 mm (E6.5)	4,87±0,73 mm (E9.5)
Implantation site thickness	2,46±0,31 mm (E6.5)	3,90±0,64 mm (E9.5)
Placental length	2,85±0,20 mm (E9.5)	6,65±0,79 mm (E16.5)
Placental thickness	2,16±0,32 mm (E9.5)	4,36±0,12 mm (E16.5)
Crown-rump length	0,50±0,21 mm (E7.5)	10,6±1,90 mm (E14.5)
Abdominal circumference	2,98±0,24 mm (E11.5)	5,89±0,23 mm (E16.5)
Antero-posterior abdominal diameter	3,42±0,24 mm (E11.5)	6,68±0,52 mm (E16.5)
Latero-lateral abdominal diameter	2,52±0,39 mm (E11.5)	5,09±0,22 mm (E16.5)
Occipital-snout length	5,23±1,1 mm (E12.5)	7,84±0,59 mm (E16.5)
Biparietal diameter	0,98±0,14 mm (E9.5)	4,84±0,13 mm (E16.5)
Subretinal space thickness	0,14±0,04 mm (E12.5)	0,21±0,01 mm (E16.5)
Antero-posterior lens diameter	0,23±0,07 mm (E12.5)	0,46±0,05 mm (E16.5)
Latero-lateral lens diameter	0,26±0,08 mm (E12.5)	0,44±0,07 mm (E16.5)
Stomach diameter	0,46±0,14 mm (E12.5)	0,75±0,24 mm (E16.5)

**Legend:** The crown-rump length (CRL) was measured as the maximum distance from the cephalic pole to the caudal pole.

The abdominal circumference (AC) was calculated by averaging the antero-posterior and latero-lateral abdominal diameters. Abdominal diameters were measured from a transverse section of the fetal abdomen at the level of the stomach and the umbilical vein.

Occipital-snout length represents the distance from the occipital prominence to the mouth.

Biparietal diameter is the distance between parietal bones and is easy to obtain by placing the electronic caliper on the hyperechogenic limit of the bones.

Measurements of placental growth included placental length and placental thickness measured at the level of umbilical vessel insertion.

**Figure 1:**
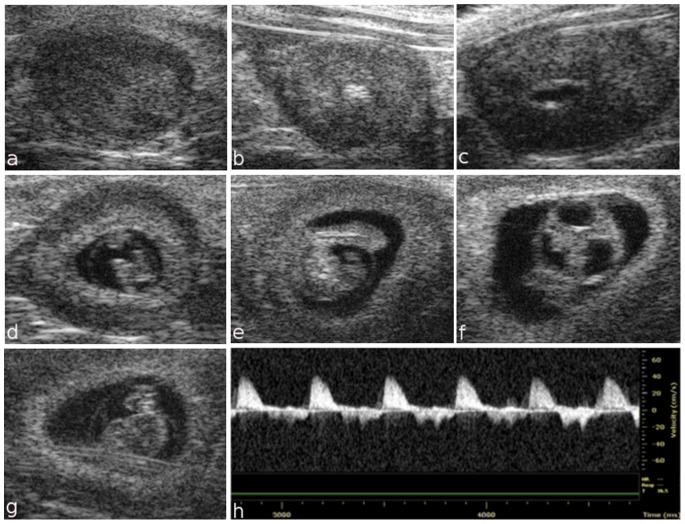
Ultrasound images of fetal development from embryonic day 5.5 to 9.5. Representative images of the implantation site at E5.5 showing the apposition of the blastocyst trophectoderm with the uterus (a). At E6.5, a small echolucent cavity containing the embryo is visualized inside the decidua (b). At E7.5, the amniotic and exocoelomic cavities and the ectoplacental cone region are discernable (c). At embryonic day 8.5, the heart, head and whole embryo are visualized (d). At E9.5, the amniotic membrane, yolk sac and cerebral ventricles are visible, while a Doppler spectral trace of ventricular inflow and outflow can be observed in the “U shaped” heart tube (e–h).

**Figure 2:**
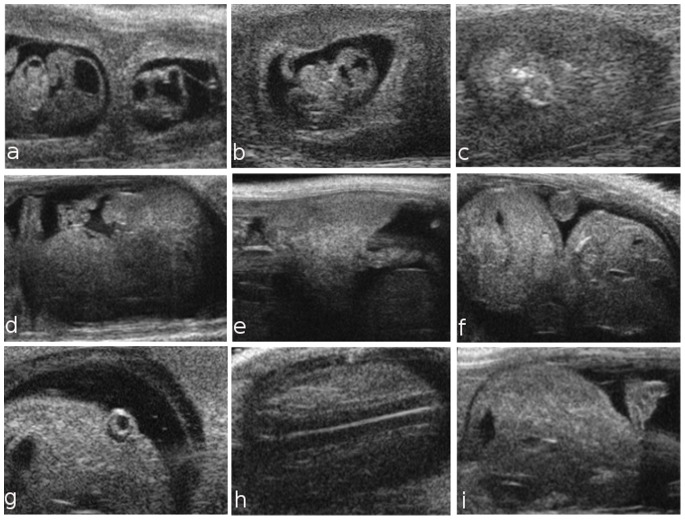
Ultrasound images of fetal development from embryonic day 10.5 to 14.5. At E10.5, it is possible to identify the umbilical cord, placenta and eyes (a, b); a sagittal view of a dead embryo is shown in (c). At embryonic day 13.5, crown-rump length (d), placental length and placental thickness (e) can be measured; the distinction between the fetal thorax and abdomen is defined (f); eyes with lens (g) and vertebral column (h) are visible. At E14.5, the occipital-snout length measurement can be reported (i).

**Figure 3:**
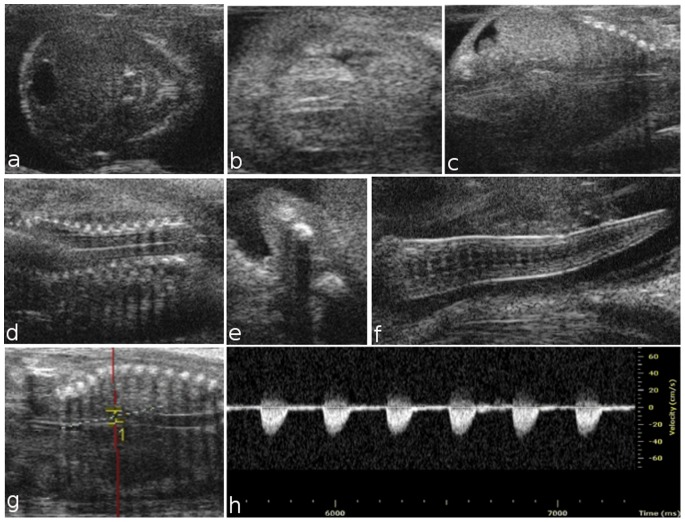
Ultrasound images of fetal development from embryonic day 14.5 to 16.5. At E14.5, progressive ossification is observed in the skull (a) and ribs (c), and the interventricular septum is completed (b). At 15.5, the development of the vertebral elements and humerus is complete (d, e). At E16.5, the curl is clearly visible (f). Dorsal aorta and corresponding Doppler spectral trace at E16.5, which exhibits a rapid upstroke and return to zero velocity during diastole (g, h).

Placental development begins in the blastocyst at E3.5, when the trophectoderm layer is segregated from the inner cell mass. At E5.5, HFUS revealed the early implantation site, which is characterized by the apposition of the trophectoderm blastocysts with the uterine epithelium (Fig. 1 a). At E6.5, the embryo was visualized inside the decidua as an echogenic ectoplacental cone region and a small echolucent cavity (Fig. 1 b). At embryonic day 7.5, the three cavities of the embryo, the amniotic, the coelomic cavity, and the ectoplacental cavity, were detected (Fig. 1 c). At E8.5, the embryo appeared to not yet have rotated into the position assumed throughout the rest of gestation, and early cardiovascular activity was visualized (Fig. 1 d). On day 9.5, the amniotic membrane, amniotic and yolk sac cavities, brain, cerebral ventricles and heart were visible (Fig. 1 e–h). At E10.5, the umbilical cord, eye lens, vitreous humor and retina were also clearly visible (Fig. 2 a, b). In the Fig. 2 c, an example of a dead embryo at E10.5 is showed. At E10.5, the open neural tube was used as the marker of brain development, while at E12.5, the brain was more clearly defined, with the mesencephalic vesicle (future aqueduct) and the third ventricle clearly visible. At embryonic day 13.5, crown-rump length could be measured, and the placenta, thorax, abdomen (Fig. 2 d–f), eye lens and vertebral column were visible (Fig. 2 g, h). At E14.5, the telencephalic vesicle and future lateral ventricles could be detected, and the occipital-snout length measurement could be made (Fig. 2 i). The choroid plexus was not routinely easily visible until E15.5, and no other individual structures could be discerned [21]. Later during embryonic development, many other structures could also be visualized, including the developing paw and forelimb, claw (Fig. 3 f), abdomen, lung, liver, kidney and blood vessels. Beginning at E14.5, the skull and ribs appear to be mineralized, and the interventricular septum is completed (Fig. 3 a–c); the same is true for vertebral elements, the humerus and the femur beginning at E15.5 (Fig. 3 d, e). At embryonic day 16.5, the dorsal aorta and corresponding Doppler spectral trace are clearly visible (Fig. 3 g, h).

Linear regression analyses (Table 3) were performed for the 3 groups. In the first group, only implantation site length, gestational sac length, gestational sac thickness and crown-rump length were significantly parameters, whereas decidual thickness and implantation site thickness remained outside the model. In the second group, gestational sac length, gestational sac thickness, placental length, crown-rump length and biparietal diameter were significant, while only placental thickness was not included in the model. In the third group, biparietal diameter, occipital-snout length, antero-posterior lens diameter, stomach diameter and antero-posterior abdominal diameter are meaningful (expressive), but placental length, placental thickness, abdominal circumference, subretinal space thickness, latero-lateral lens diameter and latero-lateral abdominal diameter were excluded from the model. The best-fit regression coefficients and standard error for the three groups of variables are shown in Table 4.

**Table 3:** Spearman’s correlation coefficient and significance of each embryonic parameter.

Embryonic Structure	Correlation Coefficient	*p*
Decidual thickness	−0,0827	0,4257
Gestational Sac Length	0,8983	<0,0001
Gestational Sac Thickness	0,8886	<0,0001
Implantation Site length	0,6234	<0,0001
Implantation SiteThickness	0,7102	<0,0001
Placental Length	0,8386	<0,0001
Placental Thickness	0,7364	<0,0001
Crown-Rump Length	0,9606	<0,0001
AbdominalCircumference	0,7426	<0,0001
Antero-PosteriorAbdominal Diameter	0,8602	<0,0001
Latero-Lateral AbdominalDiameter	0,7395	<0,0001
Occipital-snout length	0,6523	<0,0001
Biparietal Diameter	0,9485	<0,0001
Subretinal SpaceThickness	0,5559	<0,0001
Antero-Posterior LensDiameter	0,5787	<0,0001
Latero-Lateral LensDiameter	0,6255	<0,0001
Stomach Diameter	0,4617	0,0002

**Table 4:** Best-fit regression coefficients and standard error for the three groups of variables.

Embryonic day	model	Set of parameters	Coefficient	Standard error	*p*
**E7.5–E9.5**	**Group 1**	Implantation site length	0,1343	0,05011	0,0092
		Gestational sac thickness	0,1853	0,07057	0,0107
		Gestational sac length	0,4520	0,06622	<0,0001
		Crown-rump length (CRL)	0,4988	0,1061	<0,0001
		Constant	6,3475		
**E9.5–E11.5**	**Group 2**	Gestational sac thickness	0,1270	0,03938	0,0022
		Gestational sac length	0,07384	0,02222	0,0016
		Placental length	0,2023	0,05277	0,0003
		Biparietal diameter	0,2534	0,08666	0,0051
		Crown-rump length (CRL)	0,2066	0,02376	<0,0001
		Constant	7,9073		
**E12.5–E16.5**	**Group 3**	Biparietal diameter	0,7009	0,1206	
		Occipital snout length	0,1444	0,06992	
		Antero-posterior lens diameter	1,5952	0,6204	
		Stomach diameter	1,2595	0,4298	
		Antero-posterior abdominal diameter	0,4805	0,08862	
		Constant	6,7749		

## Discussion

Over the past several years, a number of authors have demonstrated the effectiveness of HFUS for staging fetal mouse gestational age and for the evaluation of morphological development in utero [22]–[25], particularly of the central nervous system, eye, and heart, in mouse embryos. HFUS appears to be a versatile tool for the detailed study of the morphogenesis of various organs. The disadvantages of HFUS are generally related to the advanced stages of gestation; some fetuses cannot be visualized or are not in an appropriate orientation or position for accurate measurements. After embryonic day 14.5, it might be difficult to obtain crown-rump length measurement, but alternative indexes of fetal size, such as head diameter, can be used at this stage. In fact, occipital-snout length and biparietal diameter of the fetal head were easily recorded in both longitudinal and transversal planes in a symmetric section of the skull. Beginning at E12.5, another anatomical structure available for measurement is the orbital diameter. This could be a useful indirect marker of brain development in mouse models because it has been reported that slow ocular growth is associated with delayed cerebral development in human fetuses [7]. Some authors showed that HFUS could affect brain development at certain frequencies [18], [22], but we determined that ultrasound performed under isoflurane anesthesia during organogenesis has no significant effects on birth weight or postnatal growth; in fact, no deaths of the anesthetized pregnant female mice occurred in our study, and embryonic or fetal death was rare. The gestation length, mean birth weight and mean number of pups per litter were not affected by ultrasound or anesthesia exposure. Additionally, the ability to determine gestational age would also allow determination of the expected time of parturition, an advantage in breeding programs with mouse strains in which delivery is difficult for the dam. The exact time of fertile mating in mice is often unknown, so the ability to monitor and measure fetal organs would allow more accurate estimation of gestational age.

We have shown that fetal and placental growth can be documented during pregnancy in mice using HFUS. We used the outbred mouse strain CD1 because it is the most widely used in basic reproductive biology studies due to the genetic variability in the experimental population. Our results can provide useful data for establishing growth curves for these structures in the strain examined, which will be a valuable tool for research in mouse pregnancy. In particular, we suggest three groups of parameters that can be used to predict the embryonic day among those parameters that can be measured at a given gestational age. HFUS is a valid methodology for the surveillance and measurement of mice embryonic development from very early stages of pregnancy. HFUS is performed in real time and is both easy and rapid. This technique is favorable for animal well being, allowing serial ultrasound examinations to be obtained on subsequent days. Moreover, the short duration of ultrasound examination avoids the risk of inducing changes in pre- and post-natal growth and development [8]. In conclusion, HFUS is an important phenotyping tool for embryonic mouse research and can be used to answer important questions in developmental biology.
