# Correction: High Frequency Ultrasound for In Vivo Pregnancy Diagnosis and Staging of Placental and Fetal Development in Mice

**DOI:** 10.1371/annotation/e710bcfd-455b-45b0-9166-eba8b5343213

**Published:** 2014-01-06

**Authors:** Adelaide Greco, Monica Ragucci, Anna Rita Daniela Coda, Alessandro Rosa, Sara Gargiulo, Raffaele Liuzzi, Matteo Gramanzini, Sandra Albanese, Sabina Pappatà, Marcello Mancini, Arturo Brunetti, Marco Salvatore

The fourth, eighth and 11th authors were incorrectly indicated as being affiliated with institutions number 2 and 3. These authors are only affiliated with institutions numbers 1 (Dipartimento di Scienze Biomediche Avanzate, Università degli studi di Napoli Federico II, Napoli, Italy) and 4 (Ceinge, Biotecnologie Avanzate, scarl, Napoli, Italy). 

